# Hypoxic Conditioned Medium from Rat Cerebral Cortical Cells Enhances the Proliferation and Differentiation of Neural Stem Cells Mainly through PI3-K/Akt Pathways

**DOI:** 10.1371/journal.pone.0111938

**Published:** 2014-11-11

**Authors:** Ming Cai, Yuehui Zhou, Bin Zhou, Shujie Lou

**Affiliations:** 1 Shanghai Key Laboratory of Human Sport Competence Development and Maintenance, Shanghai University of sport, Shanghai, China; 2 Department of Physiology, Brody school of medicine, East Carolina University, Greenville, North Carolina, United States of America; Temple University School of Medicine, United States of America

## Abstract

**Purpose:**

To investigate the effects of hypoxic conditioned media from rat cerebral cortical cells on the proliferation and differentiation of neural stem cells (NSCs) *in vitro*, and to study the roles of PI3-K/Akt and JNK signal transduction pathways in these processes.

**Methods:**

Cerebral cortical cells from neonatal Sprague–Dawley rat were cultured under hypoxic and normoxic conditions; the supernatant was collected and named ‘hypoxic conditioned medium’ (HCM) and ‘normoxic conditioned medium’ (NCM), respectively. We detected the protein levels (by ELISA) of VEGF and BDNF in the conditioned media and mRNA levels (by RT-PCR) in cerebral cortical cells. The proliferation (number and size of neurospheres) and differentiation (proportion of neurons and astrocytes over total cells) of NSCs was assessed. LY294002 and SP600125, inhibitors of PI3-K/Akt and JNK, respectively, were applied, and the phosphorylation levels of PI3-K, Akt and JNK were measured by western blot.

**Results:**

The protein levels and mRNA expressions of VEGF and BDNF in 4% HCM and 1% HCM were both higher than that of those in NCM. The efficiency and speed of NSCs proliferation was enhanced in 4% HCM compared with 1% HCM. The highest percentage of neurons and lowest percentage of astrocytes was found in 4% HCM. However, the enhancement of NSCs proliferation and differentiation into neurons accelerated by 4% HCM was inhibited by LY294002 and SP600125, with LY294002 having a stronger inhibitory effect. The increased phosphorylation levels of PI3-K, Akt and JNK in 4% HCM were blocked by LY294002 and SP600125.

**Conclusions:**

4%HCM could promote NSCs proliferation and differentiation into high percentage of neurons, these processes may be mainly through PI3-K/Akt pathways.

## Introduction

Neural stem cells (NSCs) are multipotent cells that have the capacity to self-renew and differentiate into neurons, astrocytes and oligodendrocytes [1]. They are present mainly in the developing and adult central nervous system (CNS) of mammals, including humans, where they are found specifically in two brain regions: the subventricular zone (SVZ) and subgranular zone (SGZ) of the hippocampal dentate gyrus [2]. The discovery of NSCs led to the hope that stem cell transplantation could be used to treat neurodegenerative diseases. It has been demonstrated that most NSCs are in a quiescent state under physiological conditions, but that pathological conditions, such as stroke or ischemia, could affect their proliferation and differentiation. Oxygen is a potent biochemical signaling molecule that is vital for maintaining the normal structure, function, metabolism and energy production of mammalian cells. Hypoxia affects critical cellular processes, such as survival, proliferation, apoptosis, extracellular matrix secretion, the expression of neurotrophic factors, and neuronal differentiation [3]–[6].

Hypoxia affects the cellular response of NSCs *in vivo*, which is thought to occur via two mechanisms: i) hypoxia acts directly on NSCs to affect cell signaling pathways and expression of target genes; and ii) hypoxia stimulates neurons, glial cells, and other cells that surround NSCs to secrete various paracrine factors, such as vascular endothelial growth factor (VEGF) and brain-derived neurotrophic factor (BDNF), which act synergistically to indirectly regulate the proliferation and differentiation of NSCs through the paracrine pathways [7], [8]. However, it is not clear whether and how the paracrine pathways influence the proliferation and differentiation of NSCs, which is of potential importance for the understanding of the regulation of these cells *in vivo*.

It is well known that the microenvironment that NSCs live in (the neurogenic niche) is vital for determining their fate. Previous studies have demonstrated that cells can secrete various bioactive proteins into their microenvironment whether they are *in vivo* or cultured *in vitro*. The conditioned medium from cells cultured under normoxic condition, which contains secreted proteins, plays an important role in regulating the behavior of stem cells *in vitro*, and has protective and trophic effects [9], [10]. Recent evidence has shown that conditioned medium from cells cultured under hypoxic conditions can also influence the migration, proliferation and differentiation of stem cells [6]. However, there is a lack of comparative studies about how different hypoxic concentrations of conditioned media from cerebral cortex cells influence the proliferation and differentiation of neural stem cells *in vitro*. This is of potential importance for the understanding of how the cells are regulated under different ischemia/hypoxia conditions *in vivo*, and the research regarding the underlying signal transduction mechanisms is currently insufficient.

Therefore, the purpose of this study was to determine whether different hypoxic stimulations can induce cerebral cortical cells to secrete paracrine factors (VEGF, BDNF) into conditioned media, and whether different conditioned media have different impacts on the proliferation and differentiation of NSCs *in vitro*. In addition, we examined the roles of the signal transduction pathways of PI3-K/Akt and JNK in these processes.

## Materials and Methods

### Materials and reagents

Neonatal Sprague–Dawley (SD) rats born within 24 h were purchased from the Experimental Animal Center of The Second Military Medical University (Certificate SCXK 2008–0016). All studies were conducted in accordance with the Science Research Ethics Committee of Shanghai University of Sport (No. 05–2012). Experimental protocols were approved by the Animal Care and Use Regulation of Shanghai University of Sport. All efforts were made to minimize the number of animals involved and potential of sufferings.

β-Tubulin III (β-TubIII), glial fibrillary acidic protein (GFAP), LY294002 (PI3-K inhibitor), SP600125 (JNK inhibitor), tetraethyl rhodamine isothiocyanate (TRITC)-conjugated goat anti-rat IgG, and Nestin were purchased from Sigma (St Louis, MO, USA). Neurobasal culture medium (with high glutamine), B27 supplement, and fetal bovine serum (FBS) were purchased from Gibco (Gaithersburg, MD, USA). Human basic FGF (bFGF) was purchased from Peprotech (Rocky Hill, NJ, USA). Fluorescein isothiocyanate (FITC)-conjugated anti-mouse IgG, Trizol reagent, 1st Strand cDNA Synthesis Kit, and TaqDNA polymerase were purchased from Invitrogen (Carlsbad, CA, USA). Mouse VEGF and BDNF ELISA Kit were purchased from RayBiotech (Norcross, GA, USA). Anti-VEGF, anti-BDNF, and protease inhibitor cocktail were purchased from Millipore (Bedford, MA, USA). PI3 kinase p85 antibody, phospho-PI3 kinase p85 (Tyr458)/p55(Tyr199) antibody, Akt antibody, phospho-Akt (Ser473), SAPK/JNK antibody, phospho-SAPK/JNK (Thr183/tyr185) antibody, β-Actin (13E5) rabbit mAb, and anti-rabbit IgG were purchased from Cell Signaling Technology (Beverly, MA, USA). Penicillin/streptomycin, Hoechst33258, ECL, enhanced BCA protein assay kits were purchased from Beyotime Institute of Biotechnology (Beyotime, Shanghai, P. R. China). PVDF membranes were purchased from Millipore (Bedford, MA, USA). The CO_2_ cell incubator was purchased from Thermo Scientific (Waltham, MA, USA); the Ruskinn HypOxystation was purchased from Don Whitley Scientific (West Yorkshire, England, UK); the inverted fluorescence microscope was purchased from Leica (Solms, Hesse, Germany); and the Epoch multi-volume spectrophotometer system was purchased from BioTek (Winooski, VT, USA). The GelDoc XR system and SYBR-Green PCR Master Mix were purchased from Bio-Rad (Hercules, CA, USA). The vertical electrophoresis SE300 and transfer tank were purchased from Hoefer (San Francisco, CA, USA).

### Cerebral cortical cells culture and preparation of hypoxic conditioned media

The cerebral cortex was obtained as described previously with slight modifications [11]. Briefly, under isoflurane anesthesia, the newborn SD rat born within 24 h was disinfected with 95% ethanol and decapitated immediately. The brain was dissected in a petri dish containing pre-cooling PBS. All the processes were operated on crushed ice. After removing the meninges and blood vessels, the cortex was cut into pieces and digested with 0.25% trypsin for 10–15 min at 37°C. Then cells were mechanically disaggregated with a fire-polished Pasteur pipette. The dissociated cells were centrifuged, resuspended with Neurobasal culture medium containing 2% (v/v) B27, 1% penicillin and streptomycin. After staining with Trypan Blue to assess the activity, the cells were plated at a density of 6.5×10^5^ cells/ml in a 24-well plate (previously coated with poly-L-lysine), and incubated in an atmosphere of 5% CO_2_, at 37°C. The culture medium was fully renewed at 5 d, and the cells were divided into two parts, one was put into HypOxystation with 4% or 1% O_2_, at 37°C. The cells were incubated for 6 h with different O_2_ concentrations in order to maximize the concentrations of secreted proteins. Then, the culture media were collected and centrifuged, the supernatant was named ‘hypoxic conditioned medium’ (HCM). The other part was incubated in a CO_2_ cell incubator with 5% CO_2_, at 37°C for 6 h, the culture medium was collected and centrifuged as above, and the supernatant was named ‘normoxic conditioned medium’ (NCM).

### ELISA

We detected the protein levels of VEGF and BDNF in 4% HCM, 1% HCM and NCM, using the method of double antibody sandwich BA-ELISA, according to the protocols of the manufacturer. Briefly, all samples were incubated for 2 h at room temperature (RT), in 96-well plates that were previously coated with anti-VEGF and anti-BDNF monoclonal antibodies at 4°C overnight. Then, the wells were incubated with secondary antibodies (anti-human VEGF and BDNF polyclonal antibodies) for 2 h at RT. After rinsing, anti-IgY-horseradish peroxidase (HRP) conjugate streptavidin was added into the wells and incubated for 1 h at RT. The absorbance value was read at 450 nm on a microplate reader. The protein concentrations of VEGF and BDNF in each well were determined by the standard curve.

Meanwhile, we observed the distribution of VEGF and BDNF in the cerebral cortical cells that were used to prepare the conditioned media, by immunofluorescence with VEGF and BDNF antibodies.

### Real-time RT-PCR

After collecting the conditioned media, the cells were harvested for RT-PCR, and total RNA was isolated using Trizol. cDNA was made from 5 µg total mRNA using a 1st strand cDNA synthesis kit (Invitrogen) at 25°C for 5 min, at 50°C for 60 min, and a 70°C for 15 min. Then, PCR amplification was performed for VEGF and BDNF with TaqDNA polymerase. The following primer sets were used: VEGF, forward 5′-GACAAGATGGTGAAGGTCGGT-3′, and reverse 5′- AGGGTAAGCCACTCACACACA-3′; BDNF, forward 5′-GACAAGATGGTGAAGGTCGGT-3′, and reverse 5′-CTTTGGCATCGTGGAAGGGCTC-3′. PCR was performed with the following cycles: for VEGF and BDNF, 40 cycles of 10 s at 95°C (denaturation); 30 s at 62°C (primer annealing), and 30 s at 70°C (primer extension). Reactions were performed using SYBR-Green PCR Master Mix. The levels of glyceraldehyde-3-phosphatedehydrogenase (GAPDH), as the internal control, were quantified in parallel with target genes. The ratio of fold change in expression of the mRNA of each sample was calculated by normalization of cycle threshold C(t) values of the target gene to the reference gene using the comparative C(t) (ΔΔC(t)) method [12]. Data were reported as the ΔC(t) and the average ratio of fold change in mRNA of interest corrected for reference gene. RT-PCR analysis of at least three independent cultures was performed for all experiments.

### Primary NSCs culture and proliferation of NSCs

NSCs were isolated from the cerebral cortex of SD rats that were born within 24 h. The dissociated tissues were digested with 0.25% trypsin, and the density of NSCs was adjusted to 6.5×10^5^ cells/ml. To analyze the effects of different conditioned media on the proliferation of NSCs, cells were cultured with either 4% HCM, 1% HCM, or NCM for 48 h. To analyze the roles of PI3-K/Akt and JNK pathways in NSCs proliferation, cells were cultured with 4% HCM, 0.5% (v/v) DMSO, LY294002, or SP600125 for 48 h. The two inhibitors were dissolved into DMSO to a final concentration of 10 µmol/l, before using. NSCs were identified with Nestin antibody at 48 h.

To analyze the proliferation efficiency of NSCs, the number of neurospheres was counted under an inverted microscope after NSCs were cultured for 24 h. At 48 h, the diameter of the neurospheres was measured under an inverted microscope to analyze the rate of proliferation of the NSCs [23]. Five fields in each well were randomly selected and photographed for statistical analysis of the number and diameter data. The results were calculated as the mean.

### Differentiation of NSCs

First, NSCs were cultured in Neurobasal culture medium supplemented with 2% (v/v) B27, bFGF (20 ng/ml) and 1% penicillin–streptomycin in 24-well plates for 48 h to form suspending neurospheres. Then, neurospheres were transferred to 24-well plates, previously coated with poly-L-lysine. After 3 h, the Neurobasal culture medium was replaced with different conditioned media. To analyze the effects of conditioned media on the differentiation of NSCs, cells were cultured with 4% HCM, 1% HCM, and NCM for 48 h. To analyze the roles of PI3-K/Akt and JNK pathways in NSCs differentiation, cells were cultured with 4% HCM, 0.5% (v/v) DMSO, LY294002, and SP600125 for 48 h. The two inhibitors were dissolved into DMSO to a final concentration of 10 µmol/l, before using. The double immunofluorescence staining method was performed to identify differentiated NSCs with specific molecular markers: β-TubIII antibody and GFAP antibody were respectively used to label newborn neurons and newborn astrocytes. Five fields in each well were randomly selected to photograph. The micrographs of β-TubIII+ cells, GFAP+ cells, and nuclei in the same field of each well were composited using Qwin V3 (Leica) image analyzer software to count the respective numbers. Then, the percentage of β-TubIII+ neurons and GFAP+ astrocytes among the total number of nuclei were respectively calculated.

### Immunofluorescence staining

We used the following procedures to identify NSCs and differentiated cells. The cells were fixed with 4% paraformaldehyde for 30 min at RT and then washed (0.01 mM PBS, 15 min). Then, cells were permeabilized with 0.5% TritonX-100 for 10 min. Normal goat serum (10%) was used to block non-specific binding for 1 h at 37°C. Cells were incubated at 4°C overnight with the following primary antibodies: Nestin (1∶200), β-TubIII (1∶2,000), GFAP (1∶400). Then, cells were washed (0.01 mM PBS, 20 min) and incubated in the dark for 1 h at 37°C with the appropriate secondary antibodies: FITC-conjugated goat anti-rat IgG for Nestin, GFAP; TRITC-conjugated anti-mouse IgG for β-TubIII. Nuclei were stained with Hoechst33258. An inverted fluorescence microscope was used to detect red or green fluorescence.

### Western blot analysis

For detecting the protein expression and kinase phosphorylation levels of PI3-K, Akt and JNK, the NSCs were cultured with NCM, 4% HCM, or 4% HCM supplemented with inhibitors of LY294002, and SP600125 for 48 h using the procedures described above. The collected neurospheres were washed in PBS, centrifuged, lysed in RIPA lysis buffer with protease inhibitor cocktail. For western blotting, protein samples containing an equal amount of protein (30 µg) were electrophoresed on 8% SDS-PAGE gels and transferred to PVDF membranes blocked with 5% non-fat milk powder in TBST buffer. Then the membranes were incubated overnight at 4°C with different primary antibodies, including PI3-K p85, phospho-PI3-K p85 (Tyr458)/p55 (Tyr199), Akt, phospho-Akt (Ser473), SAPK/JNK, and phospho-SAPK/JNK (Thr183/tyr185) (all 1∶1,000), for determining signal transduction events. After rinsing with TBST, the membranes were incubated with HRP-conjugated secondary antibody (1∶2000) for 1 h at RT. To visualize the immunoreactive proteins bands, an ECL kit was used according to the manufacturer's instructions. The density of each band was quantified using the GelDoc XR system.

### Statistical analysis

All values are shown as the means ±SD, each value represents the average of three independent experiments. The VEGF and BDNF data were analyzed by the Student's *t* test. The data of the amount and diameter of neurospheres, and the percentage of neurons and astrocytes were analyzed by one-way ANOVA with SPSS 17.0. The level of significance was set at p<0.05 unless otherwise indicated.

## Results

### Identification of cerebral cortical cells and neural stem cells

**Figure 1:**
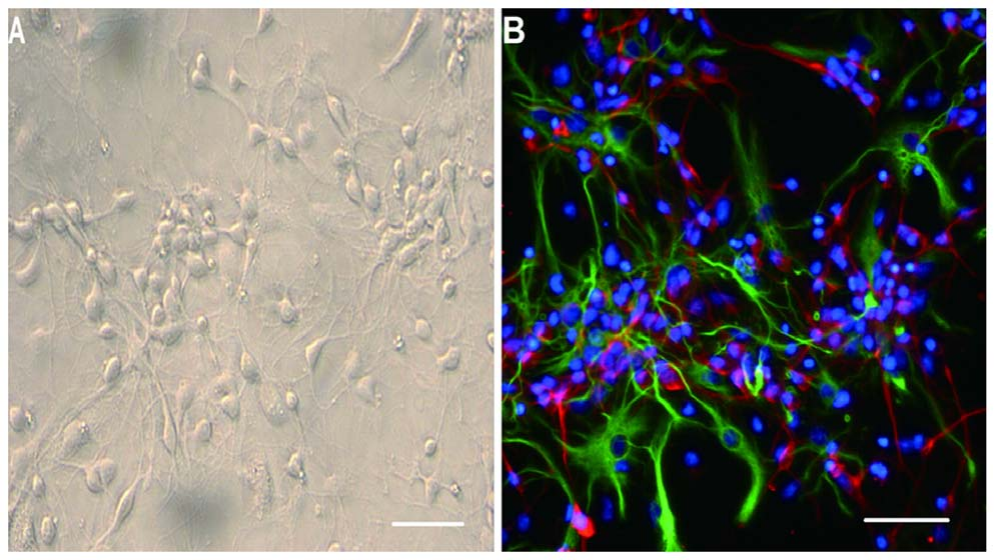
Identification of *cerebral cortical cells and* neural stem cells. The cerebral cortical cells were cultured with Neurobasal medium containing 2% B27 for 5 d; the spindly neurites grew out of the cell bodies and were observed as three-dimensional structures (A). Immunofluorescence staining showing nuclei stained blue with Hoechst33258, immunopositive neurons stained red with β-TubIII, and immunopositive astrocytes stained green with GFAP (B). Scale bar  = 200 µm.

**Figure 2:**
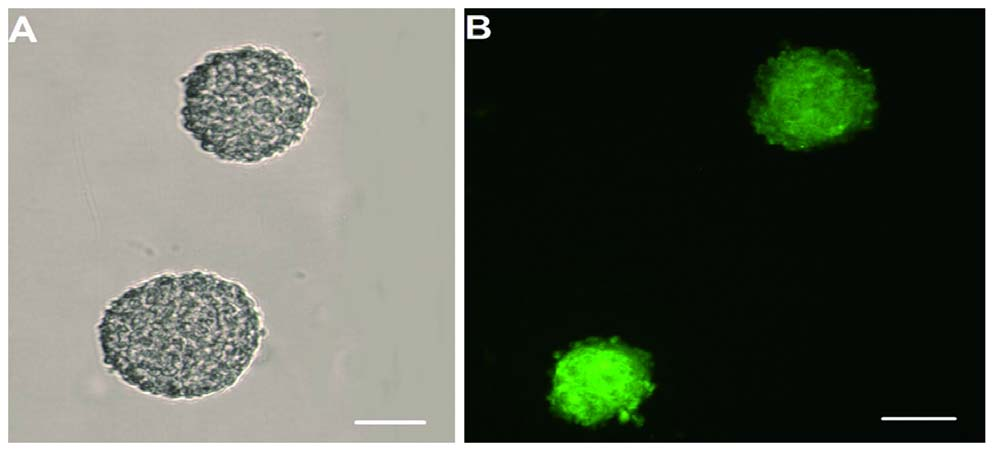
NSCs primary culture and Nestin identification. The neural stem cells were cultured with Neurobasal medium supplemented with 2% B27 and bFGF (20 ng/ml) for 48 h. The halos can be seen clearly around the round-shaped neurospheres (A). The neurospheres showed green fluorescence when they were stained with Nestin (B). Scale bar  = 200 µm.

**Figure 3:**
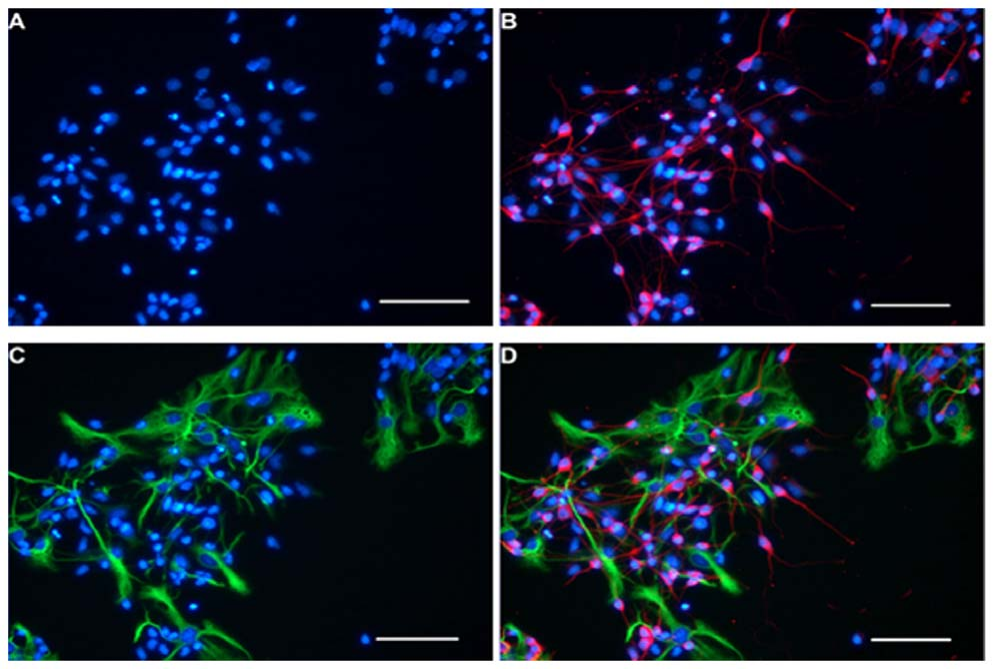
Immunofluorescence identification of differentiated NSCs. Neurons and astrocytes derived from NSCs were immunoreactive with anti-β-TubIII and anti-GFAP respectively. All of the nuclei were stained blue with Hoechst33258 (A), β-TubIII+ neurons were stained red (B), and GFAP+ astrocytes were stained green (C). Immunostaining showed that the marker β-tubIII and GFAP never co-localization at the same field (D). Scale bar  = 100 µm.

When the cerebral cortical cells were cultured with Neurobasal culture medium for 5 d to produce conditioned media, many cells changed their shapes from round (Fig. S1) to spindle or multipolar shapes. These cells also showed long neurite-like extensions that could be seen under an inverted phase contrast microscope and formed an extensive interconnecting network with adjacent neurites. This morphological progression resembled neuronal morphologies *in vitro* (Fig. 1A). Immunofluorescence staining showed that the cerebral cortical cells were composed mainly of neurons and astrocytes (Fig. 1B). When NSCs were cultured with Neurobasal culture medium supplemented with 2% (v/v) B27 and bFGF (20 ng/ml), many small suspending neurospheres could be seen under the ordinary light microscope at 24 h (Fig. S2). It was observed that the diameter of neurospheres increased with time, and the shapes became rounder (Fig. 2A). At 48 h, immunostaining showed that the cells expressed the NSCs marker, Nestin (Fig. 2B). The double immunofluorescence staining method was performed to identify differentiated progeny of NSCs, including β-TubIII+ neurons (Fig. 3B) and GFAP+ astrocytes (Fig. 3C). The β-TubIII+ and GFAP+ cells never co-localized.

### Effects of different hypoxic stimulations on the expression and secretion of VEGF and BDNF in cerebral cortical cells

**Figure 4:**
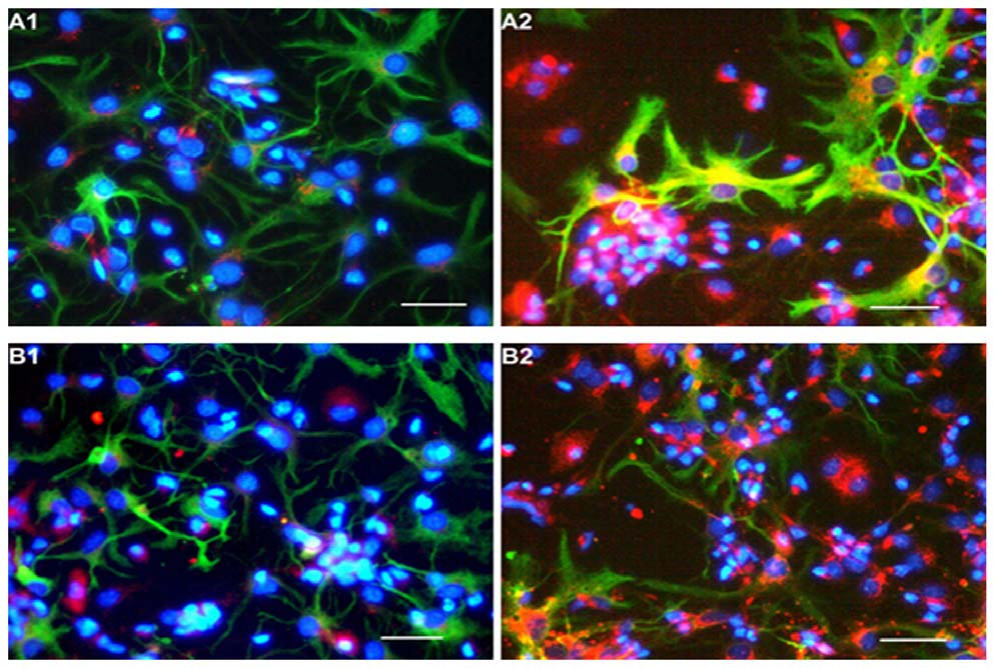
Immunofluorescence identification of VEGF and BDNF in cerebral cortical cells. Astrocytes (GFAP+) were stained green, nuclei were stained blue with Hoechst33258, both VEGF+ and BDNF+ cells were stained red. A1 and B1 represent the cells cultured with NCM, B1 and B2 represent the cells cultured with 4% HCM. Expression of VEGF and BDNF was observed in some of the astrocytes (yellow staining in the astrocytes). Scale bar  = 100 µm.

**Figure 5:**
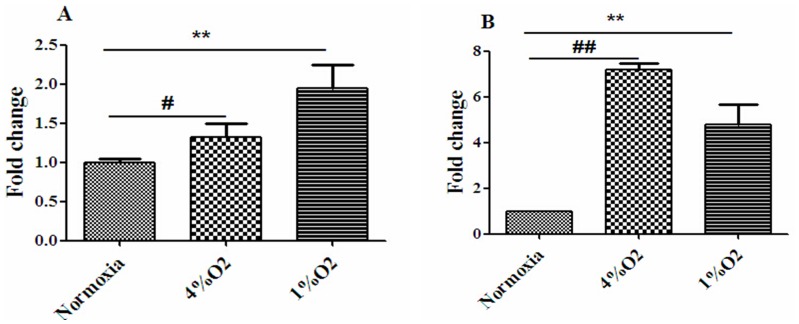
Effects of different hypoxic conditions on VEGF mRNA and BDNF mRNA levels in cerebral cortical cells. The levels of VEGF mRNA (A) and BDNF mRNA (B) in cortical cells cultured under normoxic, 1% O_2_, or 4% O_2_ conditions. Fold changes were calculated using the ΔΔ*Ct* method, and the mRNA levels of VEGF and BDNF were detected by RT-PCR. ^#^p<0.05, ^##^p<0.01, 4% O_2_ compared with normoxia; ^*^p<0.05, ^**^p<0.01, 1% O_2_ compared with normoxia (n = 3).

Immunofluorescence staining was used to observe the distribution of VEGF and BDNF in the cerebral cortical cells (Fig. 4). The expression levels of VEGF mRNA and BDNF mRNA in cerebral cortical cells were detected. RT-PCR analysis revealed that both 4% O_2_ and 1% O_2_ induced cerebral cortical cells to express more VEGF mRNA (Fig. 5A) and BDNF mRNA (Fig. 5B) as compared to normoxic stimulation.

**Figure 6:**
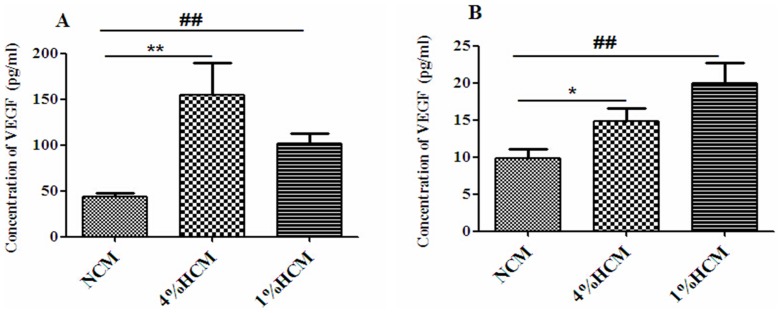
Effects of different hypoxic conditions on VEGF and BDNF secretions of cerebral cortical cells. The protein concentrations of VEGF (A) and BDNF (B) in different conditioned media were detected with ELISA. Hypoxia significantly enhanced the secretion of VEGF and BDNF compared with normoxia. ^*^p<0.05, ^**^p<0.01, 4% HCM compared with NCM; ^##^p<0.01, 1% HCM compared with NCM (n = 3).

In addition, we investigated the protein concentrations of VEGF and BDNF secreted from cerebral cortical cells into conditioned media. The results showed that the concentration of VEGF was significantly increased in the 4% HCM (154.98±34.39 pg/ml) and 1% HCM (101.32±10.87 pg/ml) compared to the NCM (44.16±3.78 pg/ml; p<0.01; Fig. 6A). In addition, there were more BDNF in the 4% HCM (14.83±1.76 pg/ml) and 1% HCM (20.02±2.66 pg/ml) than in the NCM (9.81±1.26 pg/ml; p<0.05; Fig. 6B). These results suggested that hypoxia could induce cerebral cortical cells to secret more VEGF and BDNF, especially more VEGF.

### Effects of hypoxic conditioned media on the proliferation of NSCs

**Figure 7:**
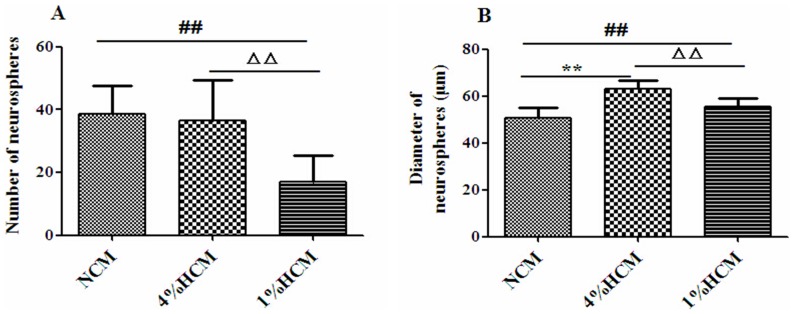
Effects of different conditioned media on the rate of proliferation of NSCs. The number of neurospheres was counted at 24 h (A), and the diameters were measured at 48 h (B). There was no difference in the number of neurospheres between 4% HCM and NCM (A). ^**^p<0.01, 4% HCM compared with NCM; ^##^p<0.01, 1% HCM compared with NCM, ^ΔΔ^p<0.05, 1% HCM compared with 4% HCM (n = 3).

NSCs were cultured with different conditioned media for 24 h in order to count the number of neurospheres. The results showed that, the 1% HCM (17.07±8.24) resulted in a significant reduction of neurospheres number compared with the NCM (38.49±9.05) and 4% HCM (36.62±12.90; p<0.01). Interestingly, there was no significant difference between the 4% HCM and NCM conditions (Fig. 7A).

NSCs were cultured for 48 h to measure the diameter of neurospheres. The results showed that the diameters of neurospheres were significantly increased in 4% HCM (62.95±3.74 µm) and 1% HCM (55.50±3.76 µm), compared to those in NCM (50.85±4.04 µm), and that the diameters of neurospheres in 4% HCM were the largest (p<0.01; Fig. 7B). At the same time, we counted the number of neurospheres and found that there was no difference when cultured for 48 h compared with that cultured for 24 h.

### Effects of hypoxic conditioned media on the differentiation of NSCs

**Figure 8:**
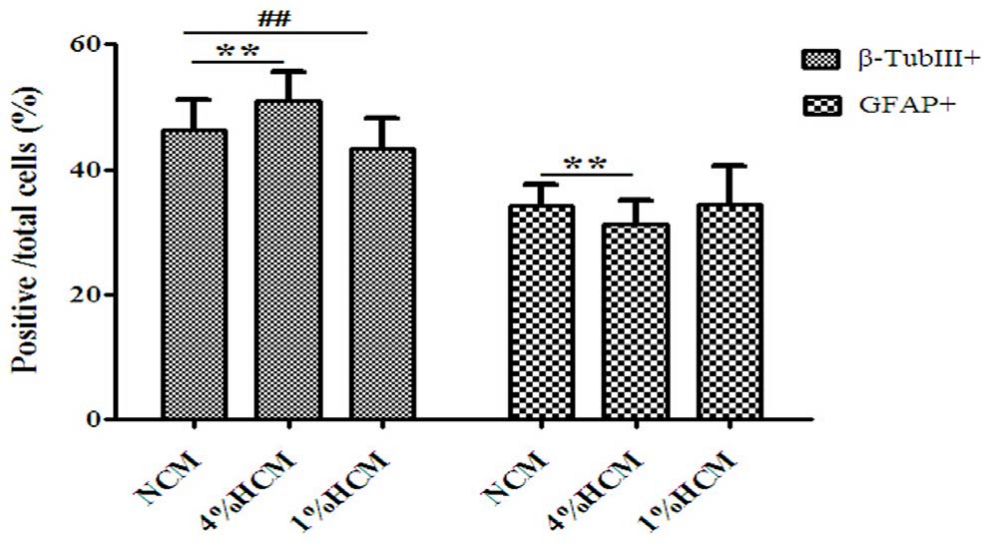
Effects of different conditioned media on the differentiation of NSCs. The highest proportion of β-TubIII+ neurons and the lowest proportion of GFAP+ astrocytes were observed with 4% HCM. The reverse results were observed with 1% HCM. ^**^p<0.01, 4% HCM compared with NCM: ^##^p<0.01, 1% HCM compared with NCM (n = 3).

Next, we evaluated the number of β-TubIII+ neurons and GFAP+ astrocytes as a percentage of the total number of cells. We found that the percentage of β-TubIII+ neurons increased significantly when cultured in 4% HCM (50.90±4.63%) compared to those cultured in 1% HCM (43.37±4.76%) and NCM (46.69±4.91%; p<0.01). Moreover, there was a significant difference in the percentage of β-TubIII+ neurons between 1% HCM and NCM (p<0.01). However, the proportion of GFAP+ astrocytes was lower in 4% HCM (31.17±3.9%) compared to those in 1% HCM (34.50±5.99%) and NCM (34.12±3.54%; p<0.01), but there was no significant difference in the percentage of GFAP+ astrocytes between 1% HCM and NCM (Fig. 8).

### Effects of signal pathway inhibitors on the proliferation of NSCs in 4% HCM

**Figure 9:**
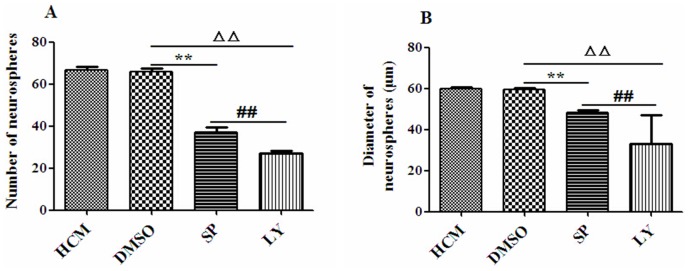
Effects of signal pathway inhibitors on the proliferation of NSCs in 4% HCM. The number of neurospheres was counted at 24 h (A), and the diameters of neurospheres were measured at 48 h (B). DMSO had no effect on NSCs proliferation. The inhibitor LY294002 resulted in a decrease of the number and diameters of neurospheres. ^**^p<0.01, SP600125 compared with DMSO; ^ΔΔ^p<0.01, LY294002 compared with DMSO, ^##^p<0.01, LY294002 compared with SP600125 (n = 3).

NSCs were cultured with 4% HCM, DMSO, SP600125 or LY294002 for 24 h, respectively. The number of neurospheres treated with SP600125 (37.00±2.28) and LY294002 (27.00±1.41) was significantly fewer in number than those cultured in 4% HCM (66.67±1.63) and DMSO (65.67±1.63; p<0.01). The results demonstrated that the fewest number of neurospheres was found in LY294002 (p<0.01). There was no significant difference between the 4% HCM and DMSO conditions (Fig. 9A).

The diameter of neurospheres was measured when the cells were cultured for 48 h. The results showed that there was no significant difference between cells cultured in DMSO (59.56±0.73 µm) and those cultured in 4% HCM (59.88±0.82 µm). However, the diameters of neurospheres from cells treated with SP600125 (48.27±1.12 µm) and LY294002 (33.24±13.92 µm) were both smaller than those that were treated with DMSO (p<0.01). Finally, the smallest diameters were observed in the LY294002 (p<0.01; Fig. 9B).

### Effects of signal pathway inhibitors on the differentiation of NSCs in 4% HCM

**Figure 10:**
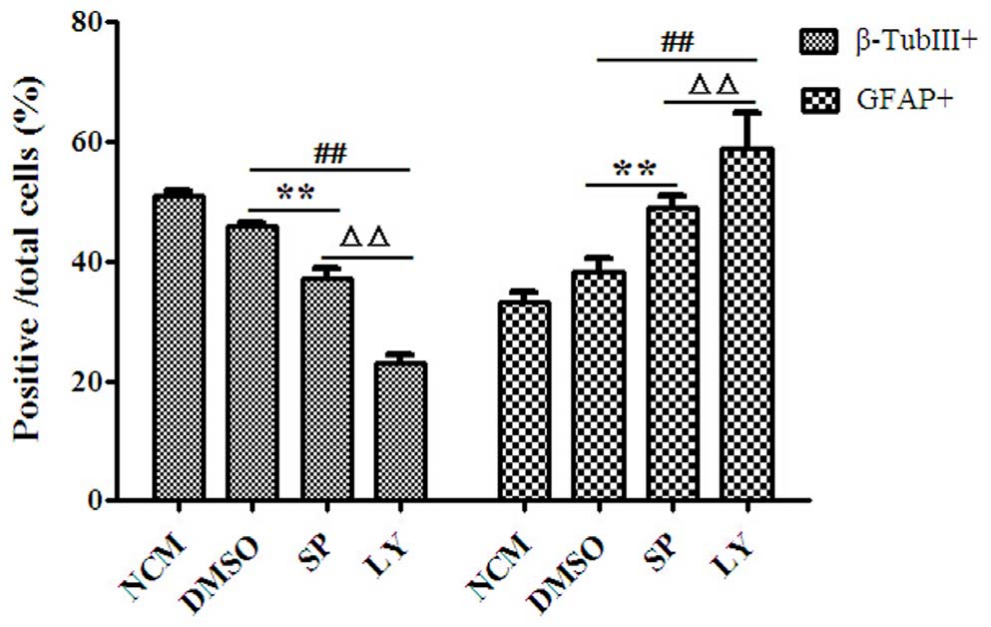
Effects of signal pathway inhibitors on the differentiation of NSCs in 4% HCM. DMSO was used as the control condition. Both LY294002 and SP600125 suppressed the ability of NSCs to differentiate into neurons. The lowest proportions of β-TubIII+ neurons and the highest proportion of GFAP+ astrocytes were observed in LY294002. ^**^p<0.01, SP600125 compared with DMSO; ^##^p<0.01, LY294002 compared with DMSO. ^ΔΔ^p<0.01, LY294002 compared with SP600125 (n = 3).

To investigate the effects of signal pathway inhibitors on NSCs differentiation, we evaluated the number of β-TubIII+ neurons and GFAP+ astrocytes as a percentage of the total number of cells. The results showed that the percentage of β-TubIII+ neurons significantly decreased in DMSO (45.85±0.73%) compared to 4% HCM (51.04±0.82%; p<0.01), which demonstrated that DMSO affected NSCs differentiation. In that case, we used DMSO as a control. The percentage of β-TubIII+ neurons in SP600125 (37.21±1.51%) and LY294002 (23.00±1.5%) were both significantly lower than those in DMSO (p<0.01), and the lowest percentage of β-TubIII+ neurons was in the LY294002 (p<0.01). However, the percentage of GFAP+ astrocytes in SP600125 (48.96±1.94%) and LY294002 (58.74±6.15%) were increased compared with that of DMSO (38.39±2.16%), with the highest percentage of GFAP+ astrocytes in the LY294002 (Fig. 10).

### Effects of 4% hypoxic conditioned medium on the phosphorylation levels of PI3-K, Akt and JNK in NSCs

**Figure 11:**
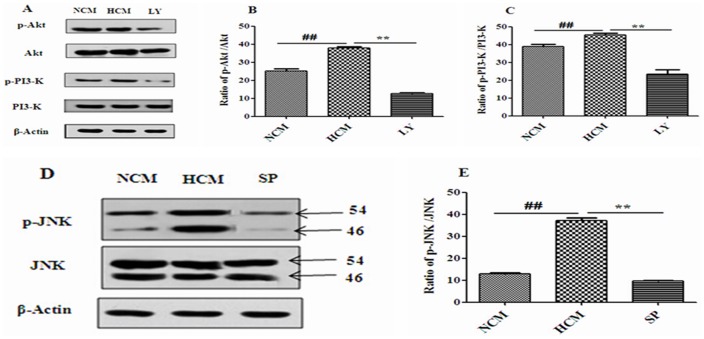
Effects of 4% HCM, LY294002 and SP600125 on the activities of PI3-K, Akt and JNK. The phosphorylation levels of Akt, PI3-K, and JNK significantly increased in HCM. High levels of p-Akt and p-PI3-K induced by HCM were blocked by LY294002 (B, C). The level of p-JNK was blocked in the presence of SP600125 (E). Representative results of the expressions of Akt/p-Akt, PI3-K/p-PI3-K (A), and JNK/p-JNK (D). ^##^p<0.01, HCM compared with NCM; ^**^p<0.01, LY and SP compared with HCM (n = 3).

NSCs were cultured with NCM, 4% HCM, or 4% HCM containing LY294002 or SP600125 for 48 h. When NSCs proliferated into neurospheres, we used Western blot to detect the phosphorylation levels of PI3-K, Akt and JNK. The findings showed that the phosphorylation levels of PI3-K, Akt and JNK increased in 4% HCM compared with NCM. Furthermore, the PI3-K inhibitor, LY294002, caused a significant reduction of phosphorylated PI3-K and Akt, and the JNK inhibitor, SP600125, also resulted in a remarkable decrease of phosphorylated JNK (Fig. 11).

## Discussion

In our studies, we selected 4% O_2_ (mild hypoxia) and 1% O_2_ (severe hypoxia) to stimulate SD rat cerebral cortical cells *in vitro*, and our findings showed that both hypoxic conditions promoted cerebral cortical cells to express VEGF and BDNF mRNA, and to induce cells to secrete VEGF and BDNF into the conditioned media as compared to the normoxic stimulations.

There is evidence to suggest that stroke or traumatic brain injury/ischemia could induce the expression of trophic factors and cytokines from NSCs, endothelial cells or other cells *in vivo* [13]. Mild hypoxic (2–5% O_2_) stimulation can also induce a significant, sustained increase in the protein levels of VEGF [14], [15] and BDNF [16] *in vitro*. However, we found that 1% O_2_ (severe hypoxia) also promoted the expression and secretion of these proteins. This result is likely to be caused by a difference in cell types or experimental conditions compared with those of other studies. Meanwhile, we found that the increased protein and expression levels of VEGF mRNA and BDNF mRNA in hypoxic conditioned media were inconsistent. We speculated it was caused by different hypoxic stimulation conditions (4%O_2_, 1% O_2_). The process of mRNA transcription and protein translation is not strictly linear, due to different regulatory mechanisms, which lead to the different detected amount of the two molecules [17].

Previous reports have shown that conditioned medium enhances the survival and migration of stem cells, as well as their proliferation and differentiation [18]. Here, we used 4% HCM and 1% HCM to culture NSCs *in vitro*, and showed that the different types of conditioned media had different effects on the efficiency and speed of NSCs proliferation. Both 4% HCM and 1% HCM increased the speed of NSCs proliferation compared with NCM, and the fastest proliferation speed of NSCs was in 4% HCM. There was a decrease in the proliferation efficiency of NSCs in 1% HCM compared with that of those in NCM, but no significant difference between the 4% HCM and NCM conditions. These results suggest that 4% HCM has a better effect on NSCs proliferation than 1% HCM. Moreover, we examined the effects of HCM on NSCs differentiation, showing that 4% HCM increased the percentage of neurons, and 1% HCM inhibited the capacity of NSCs to differentiate into neurons. Based on the above results, we found that 4% HCM from cerebral cortical cells could enhance the ability of NSCs to proliferate and differentiate into neurons, which may be due to the high levels of VEGF and BDNF in 4% HCM. These neurotrophic factors have important roles in the development and function of the CNS; they exert direct trophic and protective effects on neurons and the nervous system. They can induce the proliferation and differentiation of NSCs, and hypoxia or ischemia can influence their expressions [19].

VEGF is secreted by various cells in different brain regions, such as endothelial cells, CA1 pyramidal neurons, pyramidal neurons, astrocytes, and so on [20], [21]. VEGF secretion promotes the self-renewal of NSCs and brain development, and it has been found to confer protection from cerebral ischemia [22]. Previous reports have suggested that infusion of VEGF into the lateral ventricle increases neurogenesis by promoting survival of newborn neurons and NSCs [23], [24]. Consistently, blockade of VEGF disrupts sphere formation and neuroblasts migration [25]. VEGF exerts its biological functions through several receptors, among them VEGFR-2 (Flk-1) is believed to mediate most of the neuron-specific effects of VEGF, including neurogenesis [26], although there is recent evidence that VEGFR-1 (Flt-1) and VEGFR-3 (Flt-4) also regulate neurogenesis in the subventricular zone and dentate SGZ [27]. Hypoxia or acute ischemia can stimulate VEGF secretion through activation of hypoxia-inducible transcription factors [28], and can promote the expression of VEGF receptors [29], and VEGF could exhibit neuronal and glial protective effects [30]. BDNF is another important member of the neurotrophin family that is made in the endoplasmic reticulum and secreted from dense-core vesicles. There are two types of BDNF: pro− and mature BDNF [31], mature BDNF is crucial in the protection of the neonatal or developing brain from ischemia injury [32]. It has been shown that BDNF could activate its high-affinity tyrosine kinase receptor type B (TrkB), which was widely expressed in the adult brain, including the cortex, hippocampus, multiple brain stem [33], to activate several intracellular signaling pathways (MAPK, PI3-K, PLCγ) to play an important role in regulating the proliferation, migration, plasticity, and differentiation of NSCs in the central and peripheral nervous systems [34]–[36]. Reports have shown that hypoxia (1–3% O_2_)/ischemia increases the expression and secretion of BDNF in the forebrain, cortex and hippocampus, and promotes neurogenesis and neuronal migration to initiate repair and recovery [37]–[39]. In our study, we found that the expression and secretion of VEGF and BDNF in hypoxic stimulation were more than that in normoxic condition. Meanwhile, either in 4%HCM or in 1%HCM, the expression of VEGF was far more than BDNF. We do not know the specific reason, which needs further study. However, although we found that there were higher levels of VEGF and BDNF in 1% HCM than NCM, 1% HCM decreased the proliferation efficiency and inhibited neuronal differentiation. Presumably, the reasons for this were that the concentrations of VEGF and BDNF were lower in HCM, and 1% O_2_ was insufficient for the demand of cortical cerebral cells from neonatal rat *in vitro*. Perhapse a limited amount of energy was produced by anaerobic metabolism, leading to the production of lactic acid, oxygen radicals, and glutamate-induced excitotoxicity [13] or leading to a less efficient reprogramming process [1]. Accumulation of these substances is a sign of inadequate mitochondrial oxygenation leading to mitochondrial dysfunction and oxidative stress [40], [41], which can influence the microenvironment and the functions of paracrine factors. We will pay attention to the specific mechanisms.

However, the signal transduction mechanism of 4% HCM regulating the proliferation and differentiation of NSCs is still unproven. There is evidence that PI3-K and JNK pathways play significant roles in cell survival, proliferation, protein synthesis, and differentiation. PI3-K is a heterodimeric enzyme composed of a 110-kDa catalytic and an 85-kDa regulatory subunit [42]. The most well characterized downstream target of PI3-K is the serine-threonine kinase Akt, which transmits survival signals from growth factors to regulate a variety of cellular processes including survival, proliferation, protein translation and metabolism [43]. PI3-K can be activated by several bioactive molecules, such as NGF, VEGF and BDNF. Activation of PI3-K can promote the colocalization of Akt, and induce a conformational change in Akt that increases its phosphorylation [44]. For complete activation, Akt must be translocated to the plasma membrane, where it is phosphorylated at Ser473 and Thr308 [45]. JNK belongs to the group of mitogen-activated protein kinases (MAPKs), and is composed of two functional domains, a DNA-binding domain, located near its carboxyl-terminus and a trans-activation domain near its amino terminus [46]. VEGF and BDNF can activate JNK pathways through a mechanism that involves dual phosphorylation on the motif Thr-Pro-Tyr; this phosphorylation is mediated by the MAP kinase-kinases MKK4 and MKK7. To investigate the roles of PI3-K/Akt or JNK pathways in the proliferation and differentiation of NSCs regulated by 4% HCM, the PI3-K inhibitor LY294002 and JNK inhibitor SP600125 were used to culture NSCs *in vitro*. LY294002 is a specific inhibitor of the PI3-K signaling pathway, which can competitively inhibit the ATP binding site of the PI3-K subunit to inhibit PI3-K/Akt activation. It can significantly decrease the phosphorylation levels of PI3-K and Akt, and restrain the NSCs proliferation and differentiation into high percentage of neurons [47], [48]. SP600125 is an efficient and specific inhibitor of the JNK signaling pathway, which can significantly inhibit JNK-mediated phosphorylation of c-Jun. It can decrease the number of BrdU-labeled cells in neural precursor cells and the percentage of β-TubIII-positive cells [49], [50]. Our results show that both LY294002 and SP600125 produced a strong decrease in the efficiency and speed of proliferation, and an inhibition of NSCs differentiation into neurons in 4% HCM, and LY294002 inhibited the proliferation and differentiation of NSCs compared with SP600125. Meanwhile, the phosphorylation levels of PI3-K, Akt^Ser473^ and JNK increased when NSCs were cultured in 4% HCM compared with that of those cultured in NCM. But, when the inhibitors LY294002 and SP600125 were applied, the increased levels were significantly reduced. These results suggest that 4% HCM enhances the proliferation and differentiation of NSCs from SD rat cerebral cortical cells *in vitro* via PI3-K/Akt and JNK pathways, and that PI3-K/Akt pathways may play a major role in these processes compared with the JNK pathway.

Our data (Data S1) provide a potential explanation why hypoxia could enhance the proliferation and neuronal differentiation of NSCs. These findings represent an important advance in studying on brain injuries, such as stroke and other neurodegenerative diseases, and understanding the signal pathways in the modulation of neurogenesis. However, the exact extracellular environment, other paracrine factors in the hypoxic conditioned medium that participate in neuronal regeneration and migration after stroke have not been defined yet. The follow-up experiments that we intend to carry out in the future are to detect whether 4%HCM through VEGF and BDNF to activate PI3-K/Akt pathways, and focus on whether oxygen radicals, glutamate-induced excitotoxicity or other hazardous substances exit in 1% HCM, and whether they suppress the positive effects of VEGF and BDNF.

## Supporting Information

Figure S1 **The cerebral cortical cells were cultured immediately**. All of the cells were round, they suspended in the conditioned medium. Scale bar  = 400 µm.
(TIFF)

Figure S2 **NSCs were cultured with Neurobasal culture medium supplemented with 2% (v/v) B27 and bFGF (20 ng/ml) at 24 h.** NSCs proliferated into small neurospheres. Scale bar  = 400 µm.
(TIFF)

Data S1 **The raw data.rar.** All of the data were collected and recorded in the process of experiment, and we used them to statistical analysis and make figures.
(RAR)
