# Supplementary figures and images for: Hypoxic Conditioned Medium from Rat Cerebral Cortical Cells Enhances the Proliferation and Differentiation of Neural Stem Cells Mainly through PI3-K/Akt Pathways
Source: PLoS One. 2014 Nov 11;9(11):e111938. doi: 10.1371/journal.pone.0111938 (PMC4227679; doi:10.1371/journal.pone.0111938)

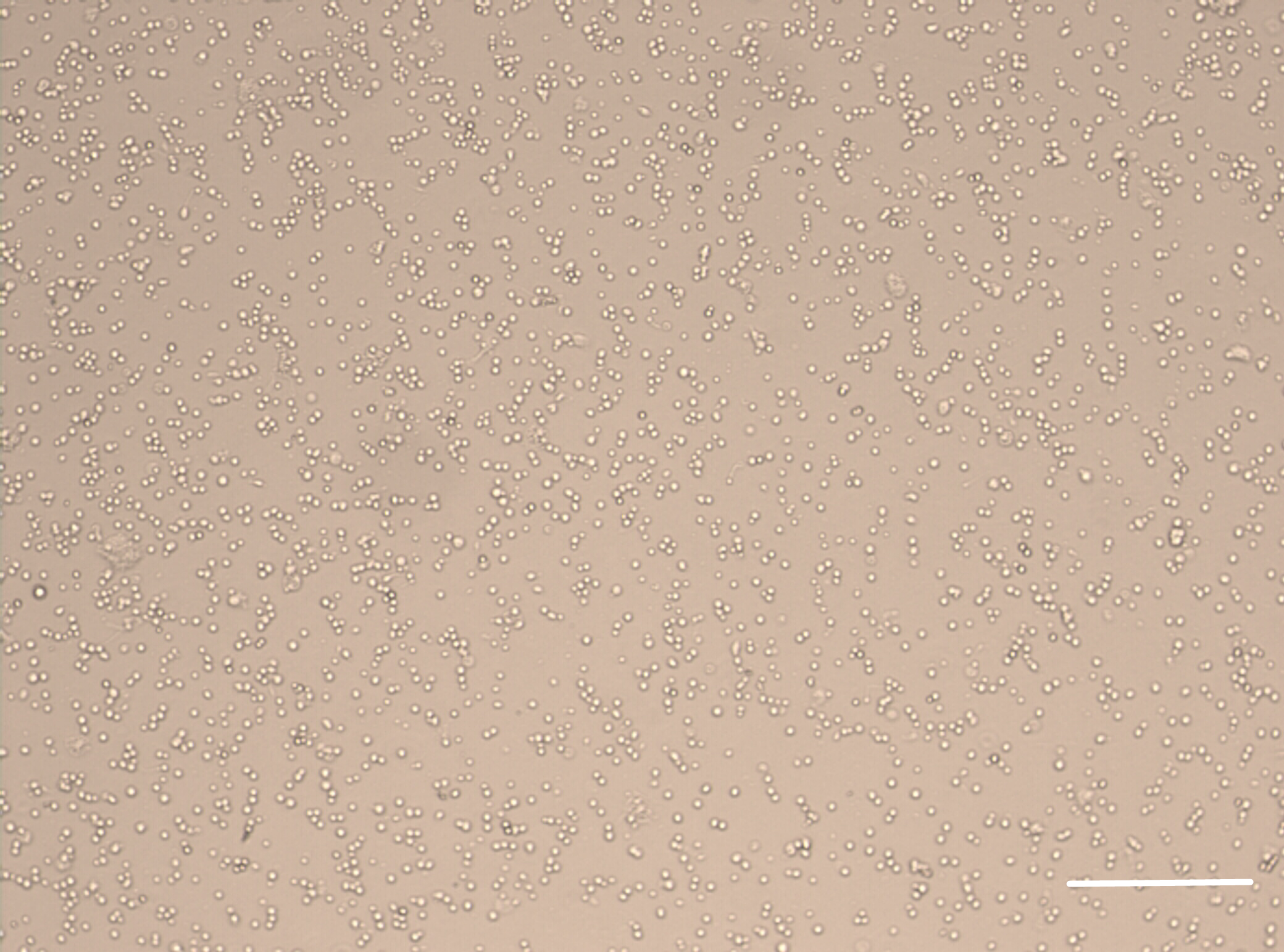

Supplement: Figure S1 — The cerebral cortical cells were cultured immediately. All of the cells were round, they suspended in the conditioned medium. Scale bar = 400 µm. (TIFF) [file pone.0111938.s001.tiff]

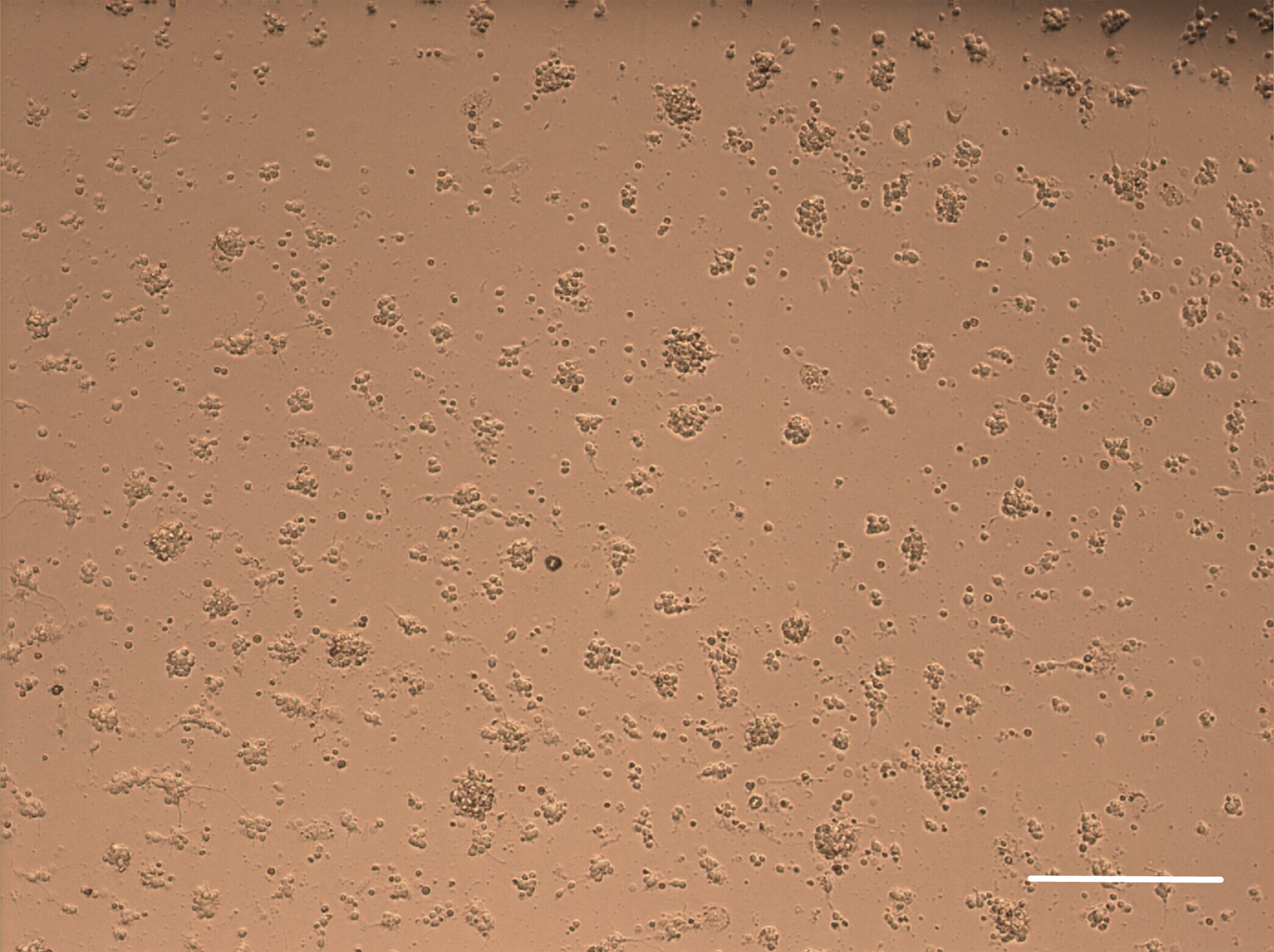

Supplement: Figure S2 — NSCs were cultured with Neurobasal culture medium supplemented with 2% (v/v) B27 and bFGF (20 ng/ml) at 24 h. NSCs proliferated into small neurospheres. Scale bar = 400 µm. (TIFF) [file pone.0111938.s002.tiff]
